# A low‐density single nucleotide polymorphism panel for brown trout (*Salmo trutta* L.) suitable for exploring genetic diversity at a range of spatial scales

**DOI:** 10.1111/jfb.15258

**Published:** 2022-11-25

**Authors:** Daniel R. Osmond, R. Andrew King, Bruce Stockley, Sophie Launey, Jamie R. Stevens

**Affiliations:** ^1^ Department of Biosciences Faculty of Health and Life Sciences, Hatherly Laboratories, University of Exeter Exeter UK; ^2^ Westcountry Rivers Trust, Rain‐Charm House Cornwall UK; ^3^ ESE, Ecology and Ecosystem Health Agrocampus Ouest INRAe Rennes France

**Keywords:** assignment, brown trout, population structure, RADseq, sea trout

## Abstract

The rivers of southern England and northern France which drain into the English Channel contain several genetically unique groups of trout (*Salmo trutta* L.) that have suffered dramatic declines in numbers over the past 40 years. Knowledge of levels and patterns of genetic diversity is essential for effective management of these vulnerable populations. Using restriction site‐associated DNA sequencing (RADseq) data, we describe the development and characterisation of a panel of 95 single nucleotide polymorphism (SNP) loci for trout from this region and investigate their applicability and variability in both target (*i.e.*, southern English) and non‐target trout populations from northern Britain and Ireland. In addition, we present three case studies which demonstrate the utility and resolution of these genetic markers at three levels of spatial separation:(a) between closely related populations in nearby rivers, (b) within a catchment and (c) when determining parentage and familial relationships between fish sampled from a single site, using both empirical and simulated data. The SNP loci will be useful for population genetic and assignment studies on brown trout within the UK and beyond.

## 1 INTRODUCTION

Central to effective ecological conservation is the understanding of genetic diversity, within and between populations of a species (Hoban *et al*., 2020). Such genetic diversity underpins the potential of a species to adapt to its local environment and to adapt to future stressors, including predicted anthropogenic climate change, changes in community structure, novel pathogens and chemical pollutants (Garner *et al*., 2020). Studies of genetic diversity within a species can also reveal cryptic structure within an apparently homogenous group (Andersson *et al*., 2017) or reveal a genetic basis for differences in life history between different components of a species that may be of ecological importance (Arostegui *et al*., 2019), and which can then inform conservation measures to safeguard this diversity.

Early population genetic studies moved from the use of allozymes, proteins of variable structure, to an emphasis on (mostly) selectively neutral loci, *e.g*., microsatellites, to delineate relationships between populations and to reveal greater genetic polymorphism than previously possible with allozymes (Hughes & Queller, 1993). In recent years, genome sequencing has become the method of choice for the study of genome‐wide variation in living organisms. Although initial studies focused on a few so‐called model organisms, recent increases in sequencing accuracy coupled with significant reductions in cost have paved the way for the application of such methods to address population‐level questions in a range of organisms, including those with little or no pre‐existing genetic resources. Nonetheless, except for organisms with very small genomes, whole genome sequencing of many individuals remains relatively expensive, and a cost‐effective alternative is to screen genomic data to identify single nucleotide polymorphisms (SNPs) (Fuentes‐Pardo & Ruzzante, 2017) that segregate with geographically distinct populations and/or phenotypic traits of interest (*e.g*., Hohenlohe *et al*., 2010). SNP markers also overcome a number of the limitations of microsatellite markers, being more frequently and evenly distributed across the genome and overcoming difficulties in standardising genotype calls between different laboratories (LaHood *et al*., 2002). Moreover, the greater density of SNPs across the genome, alongside their occurrence within coding regions with adaptive potential, has allowed the identification of loci under selection within populations (Johnston *et al*., 2014). Consequently, SNPs have become the molecular marker system of choice for cost‐effective population genetic analysis of an increasingly wide range of animal, plant and microbial taxa.

Brown trout (*Salmo trutta* L.) is a ubiquitous freshwater fish species found throughout most of Europe and with a native range stretching from Iceland in the west, Norway and Russia to the north, the Atlas mountains of North Africa to the south and Russia, Afghanistan and Pakistan in the east, as well as having readily colonised many freshwater systems around the world after introduction for recreational angling (Elliott, 1989). During the last glacial maximum (LGM) the rivers of southern England and northern continental Europe formed tributaries of a larger Channel river, draining westwards into the Bay of Biscay (Menot *et al*., 2006). Previous studies have demonstrated that this historic geography has influenced the genetic structure of brown trout across northern Europe, with catchments differentially recolonised from a number of refugial populations in southern Europe (Hamilton *et al*., 1989). As a result of these differences in the origins of recolonisation, subsequent local adaptation, highly variable life histories and the high fidelity of homing by anadromous individuals to natal rivers, there exists a marked degree of genetic structuring of brown trout populations, both within and between catchments (Griffiths *et al*., 2009; McKeown *et al*., 2010). Understanding relatedness between populations is key to effective management of this ecologically and economically valuable species (Caudron *et al*., 2006; Waples & Hendry, 2008) and can reveal impacts of anthropogenic activities on populations (Paris *et al*., 2015).

Here, we present the development of a low‐density SNP panel, using loci identified from restriction‐associated sequencing (RADseq) of trout from English Channel rivers. We then examine the effectiveness of this panel to explore patterns of genetic diversity and structure using populations from within the English Channel area as well as populations outside of this region. We then explore three case studies using this panel to examine (a) structure and genetic diversity within a single catchment relating to population fragmentation, (b) structure and genetic diversity between small proximate coastal catchments and (c) the utility of this SNP panel in identifying full‐sib family structure within a population and comparing the results to those obtained using 18 microsatellite markers.

## 2 MATERIALS AND METHODS

### 2.1 Ethical statement

The work represented here did not require ethics approval.

### 2.2 RADseq

A pooled RADseq approach (Delord *et al*., 2018) was used for SNP discovery, using a sample of 264 fish from 61 southern British rivers, 25 French rivers and 3 French hatchery stocks. Briefly, DNA was extracted from fin clips using Qiagen Blood and Tissue kits (Qiagen,Manchester, UK), quantified using Qubit dsDNA HS assays (Life Technologies, Renfrew, UK) and then combined into 20 pools (Supporting Information Table S1) based on existing knowledge of genetic structure between these populations (King *et al*., 2020, 2016; Quéméré *et al*., 2016). DNA was then digested with *Sbf*l, purified using AmpureXP magnetic beads (Beckman Coulter, High Wycombe, UK), and phased P1 adaptors were ligated onto each fragment. Digested DNA was fragmented to an average size of 400 bp, blunt ends repaired and adenylated prior to P2 adaptor ligation. The libraries were PCR amplified for 14 cycles prior to 250 bp PE sequencing on an Illumina HiSeq 2500 (Illumina, San Diego, CA, USA).

### 2.3 SNP discovery and filtering

Adaptor sequences and phasing were removed using cutadapt v2.5 (Martin, 2011) and custom scripts. Stacks v2.41 (Rochette & Catchen, 2017) was used to demultiplex and trim reads to 150 bp. RAD loci were built *de novo* using optimised parameters, following Paris *et al*. (2017). SNP discovery was carried out using the populations module, filtering for missing data, allele frequency and retaining only RAD loci with a single bi‐allelic SNP. Full details of the SNP discovery pipeline are provided in Supporting Information.

### 2.4 Non‐RADseq loci

To the RADseq‐derived loci the authors added sequence from three additional genomic regions – a three base‐pair indel polymorphism and two additional SNPs [a non‐synonymous substitution in exon 2 of the *vestigial‐like family member 3* (*vgll3*) gene and a C/G polymorphism in an intron of the *metallothionein B* (*metB*) gene]. Full details for these additional loci are given in Supporting Information. Hereafter, all loci are referred to as SNPs for clarity, including the three base‐pair indel.

### 2.5 SNP panel design

A randomly selected sub‐set of 1070 filtered RAD loci were aligned to the brown trout reference genome (Hansen *et al*., 2021) using the NCBI blastn portal (https://blast.ncbi.nlm.nih.gov/Blast.cgi). This step retained only loci that aligned with a > 99% identity score to a single linkage group (LG) and used the Genome Browser utility of SalmoBase (https://salmobase.org, Samy *et al*., 2017) to record genomic location, whether each RAD locus was within a coding or non‐coding region, and whether coding loci spanned introns, exons or both. Loci were then ranked, with preference for high‐match accuracy, singular full‐length hits and high heterozygosity. Flanking sequence data for 159 RADseq‐derived and 3 non‐RADseq loci were submitted to a commercial assay design platform (Fluidigm D3) for primer design and synthesis. These candidate loci were tested using the Fluidigm EP1 system, using an initial test panel of template DNAs from trout originating from multiple catchments. Loci that failed to amplify reliably or lacked one of the homozygous genotype clusters were exchanged for alternative assays to produce a panel of 95 reliable SNPs.

### 2.6 Population screening

To understand and validate the effectiveness of the SNP panel, we screened DNA from trout from four English (Taw, Tamar, Frome and Dour) and four French (Bresle, Sée, Touques and Flèche) rivers that flow into the English Channel, together with trout samples from three non‐Channel rivers from Britain and Ireland (Wear: Northumbria, northeast England; Burn of Arisdale: Yell, Shetland, northern Scotland, and Avoca: Co. Wicklow, southeast Ireland) (Table 1). Where possible, fish aged 1 or older were sampled to reduce the chances of collecting potentially related individuals. For British and Irish trout, DNA was extracted from adipose fin clips using the Hotshot method of Truett *et al*. (2000). For French fish, DNA was extracted from adipose fin clips using NucleoSpin 96 Tissue kits (Macherey–Nagel) following the manufacturer's protocol. SNP genotyping was undertaken using 96.96 Dynamic Genotyping Arrays on the Fluidigm EP1 Genotyping System and scored using the Fluidigm SNP Genotyping analysis software. Genotyping plots of each locus were visually inspected for quality of individual genotyping and clustering, and examples of scoring plots are given in Supporting Information Figure S1. Each run included two positive (individuals of known genotype) and two negative controls.

**TABLE 1:** Basic population diversity statistics for the brown trout populations sampled from eight target and three non‐target rivers

Study	Country	River	Tributary	Site	Grid ref (WGS84)	*N*	*H*_o_	*H*_e_	*F*_is_	%*P*
Test populations	Ireland	Avoca	Derry Water	Annacurra	52.841506, −6.355095	20	0.331	0.328	−0.036	92.6
	Scotland	Burn of Arisdale	Main river	US B9081 bridge	60.515434, −1.119758	24	0.287	0.287	−0.006	89.5
	England	Wear	Linburn Beck	Wear	54.669889, −1.769833	20	0.297	0.301	−0.012	93.7
	England	Taw	Bentwitchen Stream	Mines Bridge	51.080966, −3.802596	24	0.342	0.351	0	95.8
	England	Tamar	Main river	Gunnislake Weir	50.519114, −4.206411	24	0.359	0.362	−0.002	97.9
	England	Frome	Main river	East Stoke	50.680184, −2.183617	24	0.322	0.33	−0.01	95.8
	England	Dour	Main river	Pencester Gardens	51.127563, 1.314610	24	0.32	0.322	−0.014	93.7
	France	Flèche	Main river	St Méen	48.574090, −4.261696	24	0.297	0.291	−0.044	91.6
	France	Sée	Pierrezure/Dolaine	La Grande Mardelle	48.721790, −1.016039	24	0.321	0.325	0.007	92.6
	France	Touques	Pré d'Auge	Coquainvilliers	49.199324, 0.207981	24	0.316	0.326	−0.003	97.9
	France	Bresle	Main river	Beauchamps	50.004440, 1.520278	24	0.346	0.349	−0.018	95.8
Case Study 1	England	Camel	Allen	Trehannick	50.579536, −4.733045	22	0.351	0.354	0.007	98.9
	England	Camel	Main river	Pencarrow	50.614222, −4.680346	21	0.35	0.357	0.015	96.8
	England	Camel	Stannon Stream	Stannon	50.594068, −4.687227	24	0.335	0.34	0.011	98.9
	England	Camel	Brynn Stream	Prince Park	50.447575, −4.845736	24	0.34	0.343	0.001	95.8
	England	Camel	Main river	Tressarett	50.527964, −4.699724	31	0.349	0.353	0.004	96.8
	England	Camel	Delank	Delford Bridge	50.552421, −4.663411	27	0.339	0.345	0.015	94.7
Case Study 2	England	Penberth	Un‐named tributary	Crean Mill	50.064704, −5.643932	24	0.209	0.209	0.004	65.3
	England	Trevaylor	Main river	Noongallas	50.145402, −5.551227	25	0.296	0.29	−0.011	86.3
	England	Crowlas	Main river	Nancledra	50.170509, −5.508090	25	0.274	0.276	0.001	89.5
	England	Cober	Main river	Boscadjack	50.129733, −5.257485	23	0.232	0.245	0.064	75.8
Case Study 3	England	Great Stour	Main river	Ram Lane	51.179879, 0.801018	30	0.345	0.339	0.004	95.8

*Note*: Metrics are, respectively, number of sampled individuals (*N*), observed heterozygosity (*H*_o_), expected heterozygosity (*H*_e_), inbreeding coefficient (*F*_is_) and percentage polymorphism (%*P*).

Individuals that failed to yield data at more than 10% of loci were excluded from further analysis. Basic measures of genetic diversity [observed heterozygosity and expected heterozygosity (*H*_o_ and *H*_e_, respectively), inbreeding coefficient (*F*_is_) and percentage of polymorphic loci within each sample] were calculated using GenAlEx v6.502 (Peakall & Smouse, 2012, 2006) and GenoDive version 3.03 (Meirmans, 2020).

Genepop version 4.0 (Rousset, 2017) was used with default parameters to calculate pair‐wise linkage disequilibrium between loci, and heterozygosity deficiency and excess from Hardy–Weinberg equilibrium for each locus within each population. *P*‐values for linkage disequilibrium and Hardy–Weinberg deficiency and excess were corrected for false discovery rate (FDR) using the Holm–Bonferroni correction (Holm, 1979) for multiple comparisons.

Population pair‐wise values of *F*_ST_ were calculated using GenoDive v 3.03 with significance assessed by 999 permutations of genotypes among populations. Population inbreeding coefficients (*F*_IS_) were calculated using the divBasic function of the R package diveRsity v1.9 (Keenan *et al*., 2013), and significance was tested by bootstrapping the data 1000 times. Discriminant analysis of principal components (DAPC) was performed for individuals in the adegenet R package (Jombart, 2008). The optim.a.score function of adegenet was used to determine the number of principal components to retain in DAPC analysis. Isolation by distance (IBD) analysis was assessed in R using Mantel tests of linear *F*_ST_ and distance, utilising the man.rtest function in the ade4 package (Dray & Dufour, 2007) and measuring pair‐wise distances between sites using an online distance tool (http://www.daftlogic.com/projects-google-maps-distance-calculator.htm).

We explored the utility of this SNP panel, designed around trout from multiple Channel/Manche rivers (Supporting Information Table S1), to investigate locally relevant management questions at multiple spatial scales.

#### 2.6.1 Case Study 1

**FIGURE 1:**
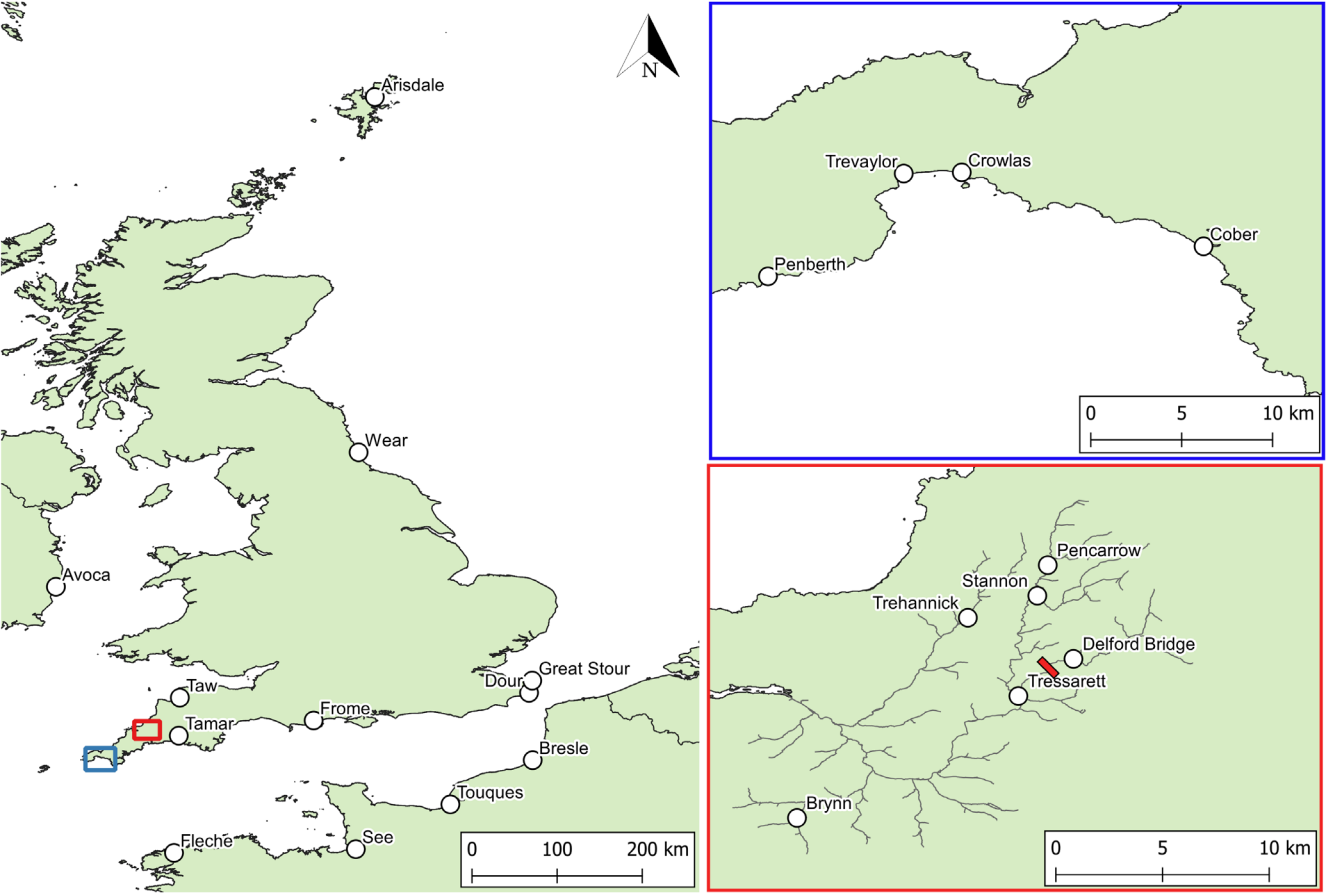
Map showing the location of rivers sampled for brown trout within the UK, France and Ireland. The left panel shows the rivers used to assess the performance of the single nucleotide polymorphisms (SNP) panel at characterising genetic parameters within and outside the target region. The top right (blue) panel shows the locations of the four sampled rivers in Mount's Bay, Cornwall (Case Study 1). The bottom right (red) panel shows the location of the sample locations in the Camel catchment (Case Study 2). The red box within the bottom right panel gives the position of the impassable De Lank quarry site

The rivers of southern Cornwall are typified by relatively small catchments inhabited by trout displaying a mosaic of genetic variation (King *et al*., 2020), with gene flow between populations maintained by straying of some anadromous individuals. We investigated the power of the SNP panel to delineate population structure between fish in these small coastal catchments. The sample consisted of 97 trout sampled from four rivers from the Mount's Bay region of southwest Cornwall (Table 1; Figure 1).

#### 2.6.2 Case Study 2

Fragmentation of river systems has been highlighted as a major driver of declines in freshwater migratory fish species (Deinet *et al*., 2020) and can have significant impacts on the diversity of fragmented populations, including the loss of allelic richness and increased genetic drift in small, isolated populations, and inbreeding depression (Coleman *et al*., 2018; Pavlova et al., 2017).

The River Camel flows from its headwaters on Bodmin Moor in eastern Cornwall over granite geology approximately 40 km to the sea at Wadebridge. The trout of this catchment appear typical of those of small coastal trout populations in southwest England, showing no obvious local (within‐catchment) sub‐structuring (King *et al*., 2016). Within the catchment, granite has been quarried from the mid‐19th century to the present day, with the De Lank tributary having been isolated from the main catchment by 300 m of granite spoil from the De Lank quarry for at least 140 years (Stanier, 1985). How this fragmentation has impacted genetic diversity of trout isolated upstream of this barrier, considered to be impassable to salmonids, was the focus of this investigation. Resident trout were sampled from six sites across the catchment, including from the De Lank upstream of the quarry spoil barrier (Table 1; Figure 1); the other five samples were from sites without any obvious barriers to fish movement.

#### 2.6.3 Case Study 3

Brown trout populations are often characterised by large numbers of closely related individuals, *i.e*., full‐sibs (Goodwin *et al*., 2016). We compared the ability of the SNP panel to assign individuals to full‐sib families with results from a panel of 18 microsatellite markers using a sample of 30 trout parr from a site on the Great Stour. We used a maximum‐likelihood method, implemented in COLONY v 2.0 (Jones & Wang, 2010) to assign sibship based on either multilocus SNP or microsatellite genotypes. Settings for COLONY were high precision, medium length run, assuming both male and female polygamy without inbreeding. To check for consistency, analyses were run twice using different random number seeds.

The ability to recover true familial relationships is dependent on the allelic diversity within the sample of individuals analysed (Hansen & Jensen, 2005). We tested the power of both the microsatellite and SNP panels to recover true full‐sib relationships and to establish whether unrelated individuals were falsely classified as full sibs. HYBRIDLAB (Nielsen *et al*., 2001) was used to simulate genotypes. To test whether the SNP panel would falsely classify unrelated individuals as full sibs, we simulated genotypes for 30 unrelated individuals (the same population genotype data were provided as the input for both “parent 1” and “parent 2” in the HYBRIDLAB interface – Nielsen *et al*., 2001) and analysed the data in COLONY using the same settings as given above. To test the ability of the SNP panel to correctly elucidate full‐sib relationships, the data set for the Great Stour population was arbitrarily split into “male” and “female” groups (15 fish in each). We simulated four full‐sib families of known parentage comprising 2, 5, 10 and 15 individuals using single “male” and “female” genotypes as input to HYBRIDLAB (Supporting Information Table S2). COLONY settings were high precision, medium length run assuming both male and female polygamy without inbreeding and a 0.25 probability that parental fish were included in the “male” and “female” data.

## 3 RESULTS

Post process_radtag filtering resulted in the retention of 116,757,729 sequences from the 20 pooled libraries (mean = 5,837,886, s.d. = 1,769,207). RAD loci were built using optimised parameters (*M* = 1, *n* = 2, *m* = 3) using the denovo_map.pl pipeline. Preliminary data analyses showed that three population pools had either low levels of sequence coverage (GB01A) or high levels of missing data (>25%, GB01B and GB04C) and were therefore removed from all subsequent analysis.

The STACKS‐filtering process resulted in a total of 7653 RAD loci containing a single variable nucleotide. To aid primer design, loci containing the variable nucleotide in the first or last 60 bp of the sequence were removed, resulting in a final data set of 5530 loci.

A total of 1070 randomly chosen RAD sequences were BLASTed against the brown trout reference genome. A whole genome duplication event in the ancestor of salmonid species *c*. 80 MYA has resulted in high levels of gene duplication (Lien *et al*., 2016). We therefore retained only those sequences (159 in total) that aligned strongly to a single genome location.

Sequences for 159 RADseq‐derived and 3 non RADseq‐derived SNPs (Supporting Information) were submitted for primer design. Initial testing of 135 loci with trout samples from a range of British, Irish and French rivers identified several loci that did not give the expected three genotype clusters, lacked one of the homozygous genotypes or had evidence for a high frequency null allele (Supporting Information Figure S1). The final panel consisted of 95 loci (Supporting Information Table S3), comprising 94 SNPs and a single 3 bp indel. The number of loci per LG ranged from zero (seven LGs) to seven (Supporting Information Figure S2), with 26 SNPs and the indel being found in non‐coding regions, 52 in introns and 15 in exons and a single SNP in a 3′ untranslated region (Supporting Information Figure S3). The Fluidigm 96.96 Dynamic Genotyping Arrays require all assay wells to be filled, so a single poorly scoring SNP was retained to complete the 96th well when running these arrays and then removed in all subsequent analysis. In future studies this unscored SNP may be replaced by a better‐performing locus of interest, though there is at present a negligible loss of analytical power from a single missing locus.

Ninety‐four SNPs were polymorphic in at least seven populations. A single locus (Str_19673) was monomorphic in all samples other than the Flèche population. A comparison of genotypes from repeated samples gave an error rate of 0.0014% (three mismatches from 2090 allele calls). No SNPs were found to be in significant linkage after Holm–Bonferri FDR correction. Across all populations, no SNPs were found to be out of Hardy–Weinberg equilibrium. Basic measures of diversity were comparable across all 11 samples. Observed heterozygosity ranged from 0.287 (Arisdale) to 0.359 (Tamar), whereas expected heterozygosity ranged from 0.287 (Arisdale) to 0.362 (Tamar) (Table 1). The percentage of polymorphic loci was lowest in the Arisdale population, with 85 variable loci. Across all populations, overall observed heterozygosity ranged from 0.265 (Str_19673) to 0.502 (Str_15228). Population pair‐wise *F*_ST_ values ranged from 0.033 (Taw and Tamar) to 0.276 (Flèche and Arisdale) (Table 2). All pair‐wise *F*_ST_ values were found to be significant. No *F*_IS_ values were significantly different from zero.

**TABLE 2:** Pair‐wise *F*_ST_ values between trout populations sampled in French, British and Irish rivers

	Taw	Tamar	Frome	Dour	Flèche	Sée	Touques	Bresle	Arisdale	Avoca	Wear
Taw	0										
Tamar	0.033	0									
Frome	0.098	0.096	0								
Dour	0.103	0.067	0.107	0							
Flèche	0.168	0.156	0.221	0.21	0						
Sée	0.097	0.085	0.095	0.109	0.147	0					
Touques	0.07	0.067	0.077	0.073	0.203	0.104	0				
Bresle	0.084	0.078	0.076	0.072	0.196	0.099	0.034	0			
Arisdale	0.117	0.135	0.175	0.162	0.276	0.172	0.127	0.155	0		
Avoca	0.042	0.049	0.121	0.109	0.188	0.112	0.067	0.09	0.102	0	
Wear	0.116	0.112	0.123	0.108	0.266	0.157	0.065	0.104	0.111	0.08	0

*Note*: All values were significant at the *P* > 0.001 level (after Holm–Bonferri false discovery rate correction).

### 3.1 Case Study 1 – population structure between trout populations in small coastal streams

**FIGURE 2:**
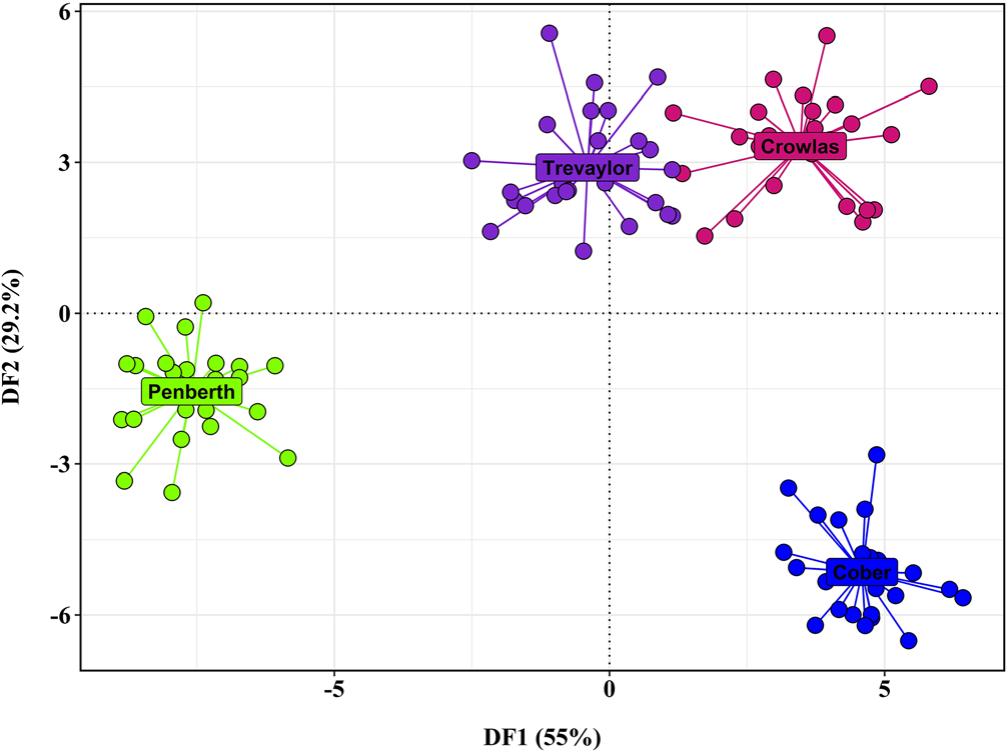
*A priori* discriminant analysis of principal components (DAPC) of trout genotypes from rivers flowing into Mount's Bay, Cornwall. Individuals are represented by individual points, with centroids for each river labelled. Discriminant function 1 (DF1) is represented by the *x* axis, and discriminant function 2 (DF2) by the *y*‐axis

No loci were found to be in significant linkage or significantly out of Hardy–Weinberg equilibrium when examined within each population inhabiting the four small streams flowing into Mount's Bay, southern Cornwall. Observed homozygosity ranged from 0.209 in Penberth to 0.296 in Trevaylor, with percentage polymorphism lowest in Penberth (65.3%) and highest in Crowlas (89.5%). Pairwise *F*_ST_ values were all significantly above zero (*p* < 0.0001) and ranged between 0.121 (Crowlas and Trevaylor) and 0.284 (Penberth and Cober) (Table 3). No *F*_IS_ values were significantly different from zero. DAPC analysis showed distinct clustering of each of the four sampled populations, with genetic distance greatest between the Penberth and Cober populations (Figure 2). These findings confirm the utility of the SNP panel for exploring genetic differentiation between closely related trout populations and the ability of the panel to robustly differentiate populations from nearby catchments.

**TABLE 3:** Pair‐wise *F*_ST_ values between rivers flowing into Mount's Bay

	Penberth	Cober	Crowlas	Trevaylor
Penberth	0			
Cober	0.284	0		
Crowlas	0.275	0.206	0	
Trevaylor	0.215	0.174	0.121	0

*Note*: All values are significant at the *P* > 0.001 level.

### 3.2 Case Study 2 – River Camel fragmentation: within catchment variation

No loci were found to be in significant linkage or out of Hardy–Weinberg equilibrium in any of the populations from the sites analysed. Observed heterozygosity was lowest in the De Lank (0.339) and greatest in the Trehannick (0.351). Percentage polymorphism varied from 94.7% (De Lank) to 98.9% (Stannon and Trehannick). No *F*_IS_ values were significantly different from zero. Pair‐wise *F*_ST_ values between sites varied between −0.001 (Pencarrow and Stannon) and 0.057 (Brynn and De Lank). *F*_ST_ values between the De Lank and each other site were all significantly greater than zero (Table 4).

**TABLE 4:** Pair‐wise *F*_ST_ values between brown trout populations sampled from the River Camel

	De Lank	Stannon	Brynn	Pencarrow	Trehannick	Tressarrett
De Lank	0					
Stannon	0.025***	0				
Brynn	0.057***	0.025***	0			
Pencarrow	0.030***	−0.001	0.021***	0		
Trehannick	0.041***	0.016***	0.011	0.017***	0	
Tressarrett	0.025***	0.006	0.022***	0.007	0.011**	0

*Note*: Significance of *F*_ST_ values, based on 999 bootstrap replicates, is indicated.

** *P* > 0.01;

*** *P* > 0.001.

**FIGURE 3:**
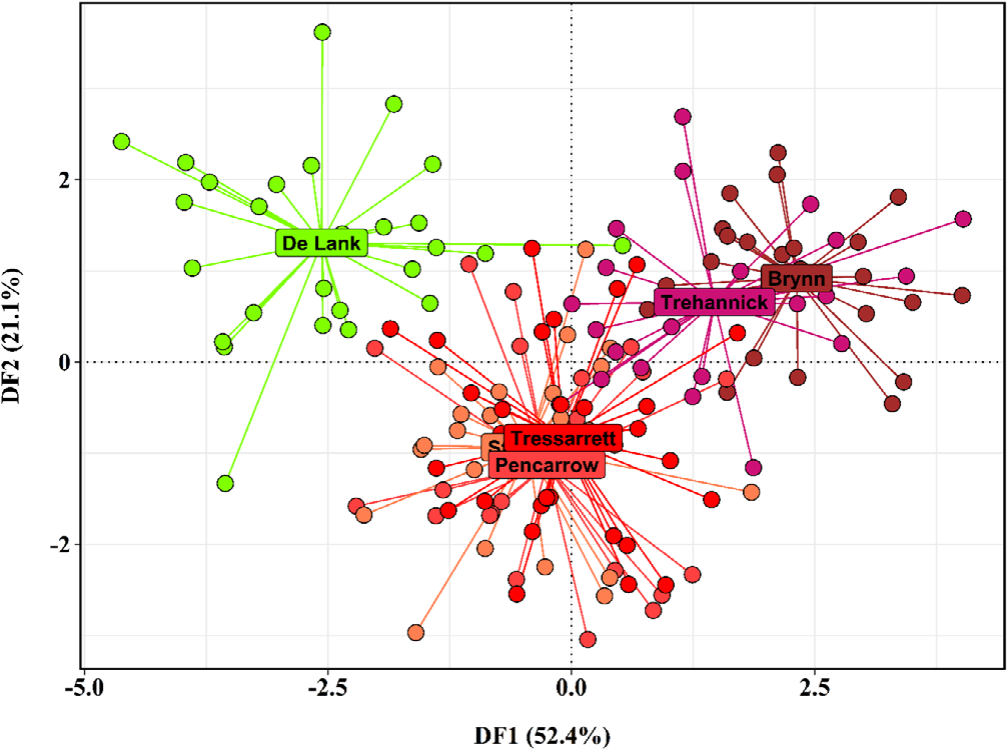
*A priori* discriminant analysis of principal components (DAPC) plot of Camel trout. Each point represents the genotype of an individual fish, with centroids for each site labelled. Discriminant function 1 (DF1) is represented by the *x* axis, and discriminant function 2 (DF2) by the *y*‐axis

**FIGURE 4:**
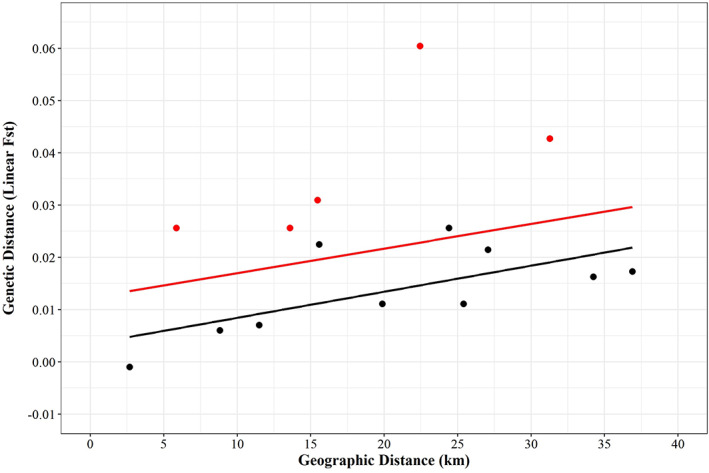
Correlation between geographic distance (km) against genetic distance (linear *F*_ST_) for the trout samples from the River Camel. The red points represent those between the De Lank and all other sites, the black points for all pair‐wise comparisons excluding the De Lank. Linear regression for all sites including the De Lank is given by the red line (*r* ^2^ = 0.321, *P* = 0.231), and linear regression for all pair‐wise sites excluding the De Lank is given by the black line (*r* ^2^ = 0.658, *P* = 0.0671)

DAPC analysis of trout in the tributaries of the Camel produced three main clusters: (a) the Trehannick and Brynn; (b) Stannon, Tressarett and Pencarrow; and (c) the De Lank (Figure 3). To examine the possibility of distance between sites driving these relationships, IBD tests were performed between (a) all sites including those sampled above the barrier on the De Lank and (b) all sites excluding the De Lank. A non‐significant positive relationship was found between linear *F*_ST_ and geographic distance between sampled populations (Figure 4; *r* ^2^ = 0.321, *P* = 0.231) and, though still non‐significant, the strength of this relationship increased when comparisons between the De Lank and other sites were excluded from the Mantel tests (Figure 4; *r* ^2^ = 0.658, *P* = 0.0671).

### 3.3 Case Study 3 – family relationships: trout in the Great Stour

Correspondence between data from 18 microsatellites and the new panel of 95 SNPs in determining full‐sib relationships for the sample of trout from the Great Stour was strong (Supporting Information Table S4). Both marker types identified six families with two or more members. The microsatellite analysis identified an additional family but with low probability of inclusion (0.766).

Simulated data showed that the microsatellite and SNP panels could reliably identify full sibship and parentage (Supporting Information Figure S4). Both data sets returned no spurious full‐sib relationships among the simulated unrelated genotypes.

## 4 DISCUSSION

Several recent studies have reported the utility of low‐density SNP arrays for the analysis of brown trout: for the elucidation of population structure in southern Europe (Saint‐Pé *et al*., 2019), and for the genetic assignment of anadromous individuals during their marine feeding phase in the North Sea (Bekkevold *et al*., 2021). This study presents a new SNP panel of 95 loci designed primarily to facilitate the investigation of population genetic diversity and structuring, and the understanding of family structure within and between brown trout populations around the English Channel. The panel provides a low‐cost, reproducible tool allowing the exploration of research questions across a hierarchy of population genetic levels: within‐population genetic parameters, population structure both within and between catchments, and assignment of family groups within a sample set. Finally, we confirm the wider utility of the panel against a range of trout populations sampled from sites across Britain, Ireland and northern France.

### 4.1 Panel performance

SNP loci are widely distributed across a genome and may be found within genes, or in significant linkage with genes or gene clusters. None of the 95 SNP loci included in the panel were significantly out of Hardy–Weinberg equilibrium, suggesting no evidence of strong selection acting on these particular markers. Similarly, the lack of significant linkage between loci in any of the screened populations suggests that all markers are independent and thereby meet the statistical assumptions required for robust downstream population genetic analysis. The panel demonstrated significant genetic differentiation between all screened populations, in accordance with previous microsatellite data reflecting genetic differences between these populations (King *et al*., 2016, 2020).

Among the lowest levels of genetic differentiation observed were those between trout populations from the Avoca, Taw and Tamar. These rivers are characterised by nutrient‐poor, acidic waters and likely have a shared phylogenetic history, recolonising the British Isles from southern refugia after the LGM (McKeown *et al*., 2010). This region of western Britain and eastern Ireland are also linked by shallow coastal seas, giving a possible source of genetic connectivity from straying of anadromous individuals feeding around the waters of the western English Channel, the Bristol Channel and southern Irish Sea. Indeed, Prodöhl *et al*. (2017) have demonstrated extensive bi‐directional movements of sea trout across the Irish Sea. Three of the trout populations analysed – Wear, Avoca and Burn of Arisdale – were outside the original target region of the SNP assay design. In comparison with the southern target populations, those from the Wear, Avoca and Arisdale represent extremes in phylogeographic distance within the British Isles; nonetheless, the panel was successful in characterising variation and structure within and between these more distant populations.

Southern Britain and southern Ireland would have remained unglaciated during the LGM and are thought to have either retained salmonid populations during this period or to have been recolonised by fish from various Atlantic refugia (Conseugra *et al*., 2002; McKeown *et al*., 2010; Finnegan *et al*., 2013). In contrast, the rivers of Shetland (Arisdale) and the east coast of England (the Wear) are thought to have had a higher input of colonisation from refugial populations of trout in the Baltic Sea and North Sea (McKeown *et al*., 2010). The variability of the loci within these non‐target populations, with polymorphism at >89.5% (Arisdale), suggests widespread variability of these SNP loci outside of English and French Channel rivers and, therefore, the potential utility of this panel in analysing trout from other non‐target rivers across Europe.

### 4.2 Between catchment structures

We tested the ability of the SNP panel to provide informative population genetic statistics and delineate structure between multiple, potentially linked catchments (Case Study 1). In the region studied – Mount's Bay, southern Cornwall – potential factors limiting gene flow include isolation by physical barriers and chemical pollutants from the industrial legacy of the region (Paris *et al*., 2015). Here we examine how these opposing drivers of population structure and gene flow affect structure and promote distinctive genetic variability within these populations.

All four sampled trout populations from the Mount's Bay region were found to be genetically distinct from one another, showing pair‐wise *F*_ST_ values significantly greater than zero and separate clustering of each of the populations in a DAPC scatterplot. Pair‐wise *F*_ST_ values are similar to those observed in a previous microsatellite‐based study of trout from small streams east of Mount's Bay (King *et al*., 2020). These results demonstrate the power of this panel of 95 SNP loci to determine relationships between populations of trout inhabiting nearby river catchments.

Observed heterozygosity and percentage polymorphism in trout populations inhabiting the Penberth and Cober are the lowest of any populations screened with the SNP panel to date, with percentage polymorphism as low as 65.3% in Penberth (King, Launey & Stevens, unpubl. data). This, alongside the relatively high pair‐wise *F*_ST_ values, suggests a lack of gene flow into and between these populations; other small coastal catchments with impassable barriers, *e.g*., the Polperro, show similarly high values of *F*_ST_ (King *et al*., 2020). The Cober terminates in its lower reaches in a natural lagoon, with connection to the sea blocked by a shingle bar – Loe Bar. The age of the formation of this natural bar is disputed, with suggestions spanning from after the last Ice Age, 5000–6000 years ago, through to more contemporary formation in the 13th century (Toy, 1934) and though flood relief channels have been installed in recent years, the routing of these – through underground culverts – likely limits the movement of anadromous trout (Vincent & Lawrence, 2020). Significantly, with the exception of occasional breaching events during severe winter storms and flood events, the Cober has been impassable for at least several hundred years. The Penberth stream is also likely impassable to anadromous trout, with a dropped weir in Penberth Cove which has been present since the development of the cove in the 19th century. These long‐standing barriers to the movement of anadromous fish (and associated gene flow) in the Penberth and Cober are likely responsible for the observed low levels of polymorphism and high *F*_ST_ with the geographically close Trevaylor and Crowlas populations. This region of Cornwall has long been exploited for metal resources. In the Cober, tin ion levels became elevated during peak extraction in the 1930s (Coard *et al*., 1983), whereas outflow from abandoned mine workings is responsible for current high levels of dissolved copper (Environment Agency, 2019); tin was also mined extensively from the region of West Penwith through which the Penberth flows (Knight & Harrison, 2013). Extraction of these metals has had significant detrimental impacts on fish and invertebrate communities, with sedimentation and chronic dissolved metal toxicity causing marked population declines (Durrant *et al*., 2011); these declines have resulted in severe population bottlenecks and have driven genetic differentiation in metal‐impacted trout populations (Paris *et al*., 2015).

The 95‐locus SNP panel presented here appears well suited to exploring this variation, as seen in several previous microsatellite‐based studies, together with additional fine levels of differentiation.

### 4.3 Within catchment structure

The brown trout populations of southwest England are typified by relatively small coastal catchments, with contemporary gene flow facilitated by the straying of anadromous individuals, with levels of genetic diversity within these small catchments often comparable to larger rivers (King *et al*., 2020). This region has, however, long been impacted by industrial processes, such as metal mining and milling, that have acted to fragment and impact resident trout populations (Jones *et al*., 2019; King *et al*., 2020; Paris *et al*., 2015). Management of these populations to conserve unique and adaptive genetic diversity must first quantify the potential impact of barriers before appropriate remedial action can be taken, making inexpensive genetic profiling of populations key to the viability of such conservation efforts.

Of the trout sampled from the River Camel, the De Lank population appeared to be the most distinct of the six sites, with pair‐wise *F*_ST_ values considerably higher than comparisons between other sampled sites; values ranged from 0.025 to 0.057. Downstream of the De Lank, the Tresarrett population, together with the Stannon and Pencarrow, clustered closely, with all *F*_ST_ values between these sites being non‐significant. Fish from the Brynn and Trehannick sites, representing tributaries closer to the mouth of the Camel, cluster together with low, non‐significant pair‐wise *F*_ST_.

Given the relative proximity of the De Lank to the other upper Camel sites, the high genetic distance between the population at this site and all other sites does not accord with the null hypothesis of a homogeneous River Camel trout population, nor an isolation‐by‐distance model of population structure. The distinctiveness of the De Lank population from the rest of the Camel accords with a hypothesis of the granite quarry acting as a barrier and driving the potential for genetic drift in the trout population isolated above the barrier. Similar patterns of post‐isolation drift have been observed in related salmonid species (Winans *et al*., 2018), and within brown trout populations isolated by hydroelectric dams (Klütsch *et al*., 2019) and barriers of chronic metal toxicity (Paris *et al*., 2015).

The quarry on the Camel has been present since at least 1880 (Stanier, 1985), giving a period of 130 years of isolation before the samples analysed in this study were collected; given an assumed generation time of 3.5 years for brown trout (Jensen *et al*., 2008), this represents approximately 35 generations. The distinctiveness of the De Lank fish is in contrast to several other studies assessing structure between isolated populations (Hoffman *et al*., 2017; Landguth *et al*., 2010) which suggest that such a small number of generations may not be sufficient to produce detectable drift and any genetic distance is likely to be small (Waples, 1998).

The results presented here have had a significant impact on the approach taken to address the De Lank Quarry conservation site. This site, as well as being a total barrier to returning adult salmonids, is having a negative impact on habitat quality downstream of the blockage (Westcountry Rivers Trust, 2022). Removing this block would in particular have a positive impact on the River Camel's salmon population. Upstream of the quarry trout have a high population density, and it was considered possible that removing the blockage would have a negative impact on the resident population via ecological niche competition with recolonising salmon (Westcountry Rivers Trust, 2022). The data presented here indicate that isolation was starting to reduce genetic diversity, with the resident trout upstream of the barrier experiencing genetic drift of relevance to the population; these trout may experience deleterious effects over following generations if population connectivity is not restored. The removal of the fish passage blockage would also have a positive impact on resident trout, by enabling gene flow mediated by anadromous sea trout from the rest of the catchment. Based on the results of this case study, a fish passage and river restoration design process at De Lank Quarry has now commenced for the benefit of both salmon and trout.

### 4.4 Family relationships

In‐river sampling of juvenile salmonid fishes for genetic analysis is often focused on just one or a few sites within a river, where juveniles (fry, parr) may originate from a very limited number of spawning adults (*e.g*., Goodwin *et al*., 2016). This can be particularly evident when juvenile fish are sampled early in the season after emerging from their spawning gravels and before they have had adequate time to disperse throughout a catchment. In such cases, samples are frequently composed of numbers of closely related juveniles, *i.e*., full‐ and half‐sibs (Goodwin *et al*., 2016; Pritchard *et al*., 2007) with retention of such individuals, especially full‐sibs, leading to potential biases in the estimation of some (but not all) population genetic parameters (Sánchez‐Montes *et al*., 2017) and misleading interpretation of population structure (Rodríguez‐Ramilo & Wang, 2012). In agreement with other studies, the panel of 95 SNP loci performed at least as well as microsatellites in assigning individuals to kin groups, even in the absence of parental genotypes (Hauser *et al*., 2011). Similarly, Hauser *et al*. (2011) found that a panel of 80 SNPs outperformed a panel of 11 microsatellites for assigning parentage in a wild population of sockeye salmon (*Oncorhynchus nerka*). In addition, using simulated data, we found no 'spurious' familial relationships, thereby increasing the confidence in the ability of their SNP panel to correctly identify full sibs within 'real”' data sets.

## 5 CONCLUSION

Here we present a low‐density SNP panel of 95 loci as a low‐cost tool for use in population genetic studies of trout (*Salmo trutta*). We find these loci to be highly polymorphic and suitable for defining population structure within the original target region – English and French rivers flowing into the Channel – and in populations around the wider British Isles. We have demonstrated the utility of this panel in three case studies: (a) an examination of relatedness between potentially linked proximate small populations; (b) an assessment of the impact of potential barriers to gene flow within a catchment; and (c) the detection of family relationships between individuals sampled from a single population. The ability to genotype large numbers of individuals across populations at relatively low cost in a short time period offers a flexibility often preferable to reduced representation sequencing approaches, while maintaining reproducibility and statistical power. The case studies also reveal some otherwise‐overlooked conservation concerns within fragmented populations, and we anticipate that this panel will be useful in future studies seeking to understand the impacts of potential stressors on genetic structure and health of threatened brown trout populations.

## Supporting information


**FIGURE S1** Example genotype scatter plots for four brown trout SNP assays genotyped on the Fluidigm EP1 platform. Str_68875 shows the three expected genotype clusters with homozygous (red and green) and heterozygous (blue) genotypes, Str_5593 lacks one of the homozygous genotype clusters, Str_59578 is an assay with a high frequency null allele and Str_10193 failed to resolve the three genotype clusters
**FIGURE S2** Number of single nucleotide polymorphisms per linkage groups for the final panel of 95 Fluidigm assays
**FIGURE S3** Position and nature (non‐coding, intronic, exonic or untranslated region) on each brown trout linkage group for the final 95 single nucleotide polymorphisms
**FIGURE S4** Results of COLONY analysis on the ability of the brown trout microsatellite and SNP panels to correctly identify familial relationships. (a) Assignment to full‐sib families and (b) assignment of parentage for 32 simulated trout genotypes. Results were identical for both marker types
**TABLE S1** Details of DNA pools used for restriction site‐associated DNA (RAD) sequencing with numbers of sequences obtained and the number retained post‐processing through the process_radtags module in STACKS v2.41 (Rochette *et al*., 2019)
**TABLE S2** Details of fullsib families simulated using HYBRIDLAB (Nielsen *et al*., 2001)
**TABLE S4** Results of COLONY analyses for 30 brown trout from the Great Stour, Kent, UK, genotyped at 18 microsatellite loci and 95 single nucleotide polymorphism assays

**TABLE S3** Details of 95 SNP assays for brown trout. Details include primer sequences, linkage group, position within linkage group, and RADtag sequence. For sequences within coding regions, we record the gene and whether the polymorphic base was found in an intron or exon. Within the RADtag sequence, the location of the polymorphic base is denoted by squared brackets *i.e*., (*[X*/*Y*)].

## References

[jfb15258-bib-0001] Andersson, A. , Jansson, E. , Wennerström, L. , Chiriboga, F. , Arnyasi, M. , Kent, M. P. , … Laikre, L. (2017). Complex genetic diversity patterns of cryptic, sympatric brown trout (*Salmo trutta*) populations in tiny mountain lakes. Conservation Genetics, 18, 1213–1227. 10.1007/s10592-017-0972-4.

[jfb15258-bib-0002] Arostegui, M. C. , Quinn, T. P. , Seeb, L. W. , Seeb, J. E. , & McKinney, G. J. (2019). Retention of a chromosomal inversion from an anadromous ancestor provides the genetic basis for alternative freshwater ecotypes in rainbow trout. Molecular Ecology, 28, 1412–1427. 10.1111/mec.15037. 30714254

[jfb15258-bib-0003] Bekkevold, D. , Piper, A. , Campbell, R. , Rippon, P. , Wright, R. M. , Crundwell, C. , … Maltby, A. (2021). Genetic stock identification of sea trout (*Salmo trutta* L.) along the British North Sea coast shows prevalent long‐distance migration. ICES Journal of Marine Science, 78, 952–966. 10.1093/icesjms/fsaa240.

[jfb15258-bib-0004] Caudron, A. , Champigneulle, A. , & Guyomard, R. (2006). Assessment of restocking as a strategy for rehabilitating a native population of brown trout *Salmo trutta* L. in a fast‐flowing mountain stream in the northern French Alps. Journal of Fish Biology, 69, 127–139. 10.1111/j.1095-8649.2006.01156.x.

[jfb15258-bib-0005] Coard, M. A. , Cousen, S. M. , Cuttler, A. H. , Dean, H. J. , Dearing, J. A. , Eglinton, T. I. , … Simola, H. (1983). Paleolimnological studies of annually‐laminated sediments in Loe Pool, Cornwall, U.K. Hydrobiologia, 103, 185–191. 10.1007/BF00028450.

[jfb15258-bib-0006] Coleman, R. A. , Gauffre, B. , Pavlova, A. , Beheregaray, L. B. , Kearns, J. , Lyon, J. , … Sunnucks, P. (2018). Artificial barriers prevent genetic recovery of small isolated populations of a low‐mobility freshwater fish. Heredity, 120, 515–532. 10.1038/s41437-017-0008-3. 29326479 PMC5943333

[jfb15258-bib-0065] Consuegra, S., Garcia De Leaniz, C., Serdio, A., Gonzalez Morales, M., Straus, L. G., Knox, D., & Verspoor, E. (2002). Mitochondrial DNA variation in Pleistocene and modern Atlantic salmon from the Iberian glacial refugium. Molecular Ecology, 11, 2037–2048. 10.1046/j.1365-294x.2002.01592.x 12296947

[jfb15258-bib-0007] Deinet, S. , Scott_Gatty, K. , Rotton, H. , Twardek, W.M. , Marconi, V. , McRae, L. , … Berkhuysen, A. (2020). *The living planet index (LPI) for migratory freshwater fish*. The Netherlands: World Fish Migration Foundation.

[jfb15258-bib-0008] Delord, C. , Lassalle, G. , Oger, A. , Barloy, D. , Coutellec, M. , Delcamp, A. , … Petit, E. J. (2018). A cost‐and‐time effective procedure to develop snp markers for multiple species: A support for community genetics. Methods in Ecology and Evolution, 9, 1959–1974. 10.1111/2041-210X.13034.

[jfb15258-bib-0009] Dray, S. , & Dufour, A.‐B. (2007). The ade4 package: Implementing the duality diagram for ecologists. Journal of Statistical Software, 22, 1–20.

[jfb15258-bib-0010] Durrant, C. J. , Stevens, J. R. , Hogstrand, C. , & Bury, N. R. (2011). The effect of metal pollution on the population genetic structure of brown trout (*Salmo trutta* L.) residing in the river Hayle, Cornwall, UK. Environmental Pollution, 159, 3595–3603. 10.1016/j.envpol.2011.08.005. 21885173

[jfb15258-bib-0011] Elliott, J. M. (1989). Wild brown trout *Salmo trutta*: An important national and international resource. Freshwater Biology, 21, 1–5. 10.1111/j.1365-2427.1989.tb01343.x.

[jfb15258-bib-0067] Environment Agency (2019). Copper RNAG in Lower River Cober. Data available from https://environment.data.gov.uk/catchment-planning/WaterBody/GB108048001172/rnag?cycle=3&element=79

[jfb15258-bib-0064] Finnegan, A. K., Griffiths, A. M., King, R. A., Machado‐Schiaffino, G., Porcher, J.‐P., Garcia‐Vazquez, E., Bright, D., & Stevens, J. R. (2013). Use of multiple markers demonstrates a cryptic western refugium and postglacial colonisation routes of Atlantic salmon (*Salmo salar* L.) in northwest Europe. Heredity, 111, 34–43. 10.1038/hdy.2013.17 PMC3692316 23512011

[jfb15258-bib-0012] Fuentes‐Pardo, A. P. , & Ruzzante, D. E. (2017). Whole‐genome sequencing approaches for conservation biology: Advantages, limitations and practical recommendations. Molecular Ecology, 26, 5369–5406. 10.1111/mec.14264. 28746784

[jfb15258-bib-0013] Garner, B. A. , Hoban, S. , & Luikart, G. (2020). IUCN red list and the value of integrating genetics. Conservation Genetics, 21, 795–801. 10.1007/s10592-020-01301-6.

[jfb15258-bib-0014] Goodwin, J. C. A. , King, R. A. , Jones, J. I. , Ibbotson, A. , & Stevens, J. R. (2016). A small number of anadromous females drive reproduction in a brown trout (*Salmo trutta*) population in an English chalk stream. Freshwater Biology, 61, 1075–1089. 10.1111/fwb.12768.

[jfb15258-bib-0015] Griffiths, A. M. , Koizumi, I. , Bright, D. , & Stevens, J. R. (2009). A case of isolation by distance and short‐term temporal stability of population structure in brown trout (*Salmo trutta*) within the river dart, Southwest England. Evolutionary Applications, 2, 537–554. 10.1111/j.1752-4571.2009.00092.x. 25567897 PMC3352451

[jfb15258-bib-0016] Hamilton, K. E. , Ferguson, A. , Taggart, J. B. , Tomasson, T. , Walker, A. , & Fahy, E. (1989). Post‐glacial colonization of brown trout, *Salmo trutta* L.: Ldh‐5 as a phylogeographic marker locus. Journal of Fish Biology, 35, 651–664. 10.1111/j.1095-8649.1989.tb03017.x.

[jfb15258-bib-0017] Hansen, M. M. , & Jensen, L. F. (2005). Sibship within samples of brown trout (*Salmo trutta*) and implications for supportive breeding. Conservation Genetics, 6, 297–305. 10.1007/s10592-004-7827-5.

[jfb15258-bib-0018] Hansen, T. , Fjelldal, P. G. , Lien, S. , Smith, M. , Corton, C. , Oliver, K. , … Blaxter, M. (2021). The genome sequence of the brown trout, *Salmo trutta* Linnaeus 1758. Wellcome Open Research, 6, 108. 10.12688/wellcomeopenres.16838.1. 34632087 PMC8488904

[jfb15258-bib-0019] Hauser, L. , Baird, M. , Hilborn, R. , Seeb, L. W. , & Seeb, J. E. (2011). An empirical comparison of SNPs and microsatellites for parentage and kinship assignment in a wild sockeye salmon (*Oncorhynchus nerka*) population: Analytical approaches. Molecular Ecology Resources, 11, 150–161. 10.1111/j.1755-0998.2010.02961.x. 21429171

[jfb15258-bib-0020] Hoban, S. , Bruford, M. , D'Urban Jackson, J. , Lopes‐Fernandes, M. , Heuertz, M. , Hohenlohe, P. A. , … Laikre, L. (2020). Genetic diversity targets and indicators in the CBD post‐2020 global biodiversity framework must be improved. Biological Conservation, 248, 108654. 10.1016/j.biocon.2020.108654.

[jfb15258-bib-0021] Hoffman, J. R. , Willoughby, J. R. , Swanson, B. J. , Pangle, K. L. , & Zanatta, D. T. (2017). Detection of barriers to dispersal is masked by long lifespans and large population sizes. Ecology and Evolution, 7, 9613–9623. 10.1002/ece3.3470. 29187994 PMC5696434

[jfb15258-bib-0022] Hohenlohe, P. A. , Bassham, S. , Etter, P. D. , Stiffler, N. , Johnson, E. A. , & Cresko, W. A. (2010). Population genomics of parallel adaptation in threespine stickleback using sequenced RAD tags. PLoS Genetics, 6, e1000862. 10.1371/journal.pgen.1000862. 20195501 PMC2829049

[jfb15258-bib-0023] Holm, S. (1979). A simple sequentially rejective multiple test procedure. Scandinavian Journal of Statistics, 6, 65–70.

[jfb15258-bib-0024] Hughes, C. R. , & Queller, D. C. (1993). Detection of highly polymorphic microsatellite loci in a species with little allozyme polymorphism. Molecular Ecology, 2, 131–137. 10.1111/j.1365-294X.1993.tb00102.x. 8167848

[jfb15258-bib-0025] Jensen, L. F. , Hansen, M. M. , Pertoldi, C. , Holdensgaard, G. , Mensberg, K.‐L. D. , & Loeschcke, V. (2008). Local adaptation in brown trout early life‐history traits: Implications for climate change adaptability. Proceedings of the Royal Society B: Biological Sciences, 275, 2859–2868. 10.1098/rspb.2008.0870. PMC2605839 18755673

[jfb15258-bib-0026] Johnston, S. E. , Orell, P. , Pritchard, V. L. , Kent, M. P. , Lien, S. , Niemelä, E. , … Primmer, C. R. (2014). Genome‐wide SNP analysis reveals a genetic basis for sea‐age variation in a wild population of Atlantic salmon (*Salmo salar*). Molecular Ecology, 23, 3452–3468. 10.1111/mec.12832. 24931807

[jfb15258-bib-0027] Jombart, T. (2008). Adegenet: A R package for the multivariate analysis of genetic markers. Bioinformatics, 24, 1403–1405. 10.1093/bioinformatics/btn129. 18397895

[jfb15258-bib-0028] Jones, J. , Börger, L. , Tummers, J. , Jones, P. , Lucas, M. , Kerr, J. , … Garcia de Leaniz, C. (2019). A comprehensive assessment of stream fragmentation in Great Britain. Science of the Total Environment, 673, 756–762. 10.1016/j.scitotenv.2019.04.125. 31003103

[jfb15258-bib-0062] Jones, O. R., & Wang, J. (2010). COLONY: a program for parentage and sibship inference from multilocus genotype data. Molecular Ecology Resources, 10(3), 551–555. Portico. 10.1111/j.1755-0998.2009.02787.x 21565056

[jfb15258-bib-0029] Keenan, K. , McGinnity, P. , Cross, T. F. , Crozier, W. W. , & Prodöhl, P. A. (2013). diveRsity: An R package for the estimation and exploration of population genetics parameters and their associated errors. Methods in Ecology and Evolution, 4, 782–788. 10.1111/2041-210X.12067.

[jfb15258-bib-0030] King, R. A. , Hillman, R. , Elsmere, P. , Stockley, B. , & Stevens, J. R. (2016). Investigating patterns of straying and mixed stock exploitation of sea trout, *Salmo trutta*, in rivers sharing an estuary in South‐West England. Fisheries Management and Ecology, 23, 376–389. 10.1111/fme.12181.

[jfb15258-bib-0031] King, R. A. , Stockley, B. , & Stevens, J. R. (2020). Small coastal streams—Critical reservoirs of genetic diversity for trout (*Salmo trutta* L.) in the face of increasing anthropogenic stressors. Ecology and Evolution, 10, 5651–5669. 10.1002/ece3.6306. 32607181 PMC7319166

[jfb15258-bib-0032] Klütsch, C. F. C. , Maduna, S. N. , Polikarpova, N. , Forfang, K. , Aspholm, P. E. , Nyman, T. , … Hagen, S. B. (2019). Genetic changes caused by restocking and hydroelectric dams in demographically bottlenecked brown trout in a transnational subarctic riverine system. Ecology and Evolution, 9, 6068–6081. 10.1002/ece3.5191. 31161019 PMC6540707

[jfb15258-bib-0033] Knight, J. , & Harrison, S. (2013). ‘A land history of men’: The intersection of geomorphology, culture and heritage in Cornwall, Southwest England. Applied Geography, 42, 186–194. 10.1016/j.apgeog.2013.03.020.

[jfb15258-bib-0034] LaHood, E. S. , Moran, P. , Olsen, J. , Grant, W. S. , & Park, L. K. (2002). Microsatellite allele ladders in two species of Pacific salmon: Preparation and field‐test results. Molecular Ecology Notes, 2, 187–190. 10.1046/j.1471-8286.2002.00174.x.

[jfb15258-bib-0035] Landguth, E. L. , Cushman, S. A. , Schwartz, M. K. , McKELVEY, K. S. , Murphy, M. , & Luikart, G. (2010). Quantifying the lag time to detect barriers in landscape genetics: Quantifying the lag time to detect barriers in landscape genetics. Molecular Ecology, 19, 4179–4191. 10.1111/j.1365-294X.2010.04808.x. 20819159

[jfb15258-bib-0036] Lien, S. , Koop, B. F. , Sandve, S. R. , Miller, J. R. , Kent, M. P. , Nome, T. , … Davidson, W. S. (2016). The Atlantic salmon genome provides insights into rediploidization. Nature, 533, 200–205. 10.1038/nature17164. 27088604 PMC8127823

[jfb15258-bib-0037] Martin, M. (2011). Cutadapt removes adapter sequences from high‐throughput sequencing reads. EMBnet. Journal, 17, 10. 10.14806/ej.17.1.200.

[jfb15258-bib-0038] McKeown, N. J. , Hynes, R. A. , Duguid, R. A. , Ferguson, A. , & Prodöhl, P. A. (2010). Phylogeographic structure of brown trout *Salmo trutta* in Britain and Ireland: Glacial refugia, postglacial colonization and origins of sympatric populations. Journal of Fish Biology, 76, 319–347. 10.1111/j.1095-8649.2009.02490.x. 20738710

[jfb15258-bib-0039] Meirmans, P. G. (2020). Genodive version 3.0: Easy‐to‐use software for the analysis of genetic data of diploids and polyploids. Molecular Ecology Resources, 20, 1126–1131. 10.1111/1755-0998.13145. 32061017 PMC7496249

[jfb15258-bib-0040] Menot, G. , Bard, E. , Rostek, F. , Weijers, J. W. H. , Hopmans, E. C. , Schouten, S. , & Damste, J. S. S. (2006). Early reactivation of European Rivers during the last deglaciation. Science, 313, 1623–1625. 10.1126/science.1130511. 16973877

[jfb15258-bib-0041] Nielsen, E. E. , Hansen, M. M. , & Bach, L. A. (2001). Looking for a needle in a haystack: Discovery of indigenous salmon in heavily stocked populations. Conservation Genetics, 2, 219–232. 10.1023/A:1012239029574.

[jfb15258-bib-0042] Paris, J. R. , King, R. A. , & Stevens, J. R. (2015). Human mining activity across the ages determines the genetic structure of modern brown trout (*Salmo trutta* L.) populations. Evolutionary Applications, 8, 573–585. 10.1111/eva.12266. 26136823 PMC4479513

[jfb15258-bib-0043] Paris, J. R. , Stevens, J. R. , & Catchen, J. M. (2017). Lost in parameter space: A road map for stacks . Methods in Ecology and Evolution, 8, 1360–1373. 10.1111/2041-210X.12775.

[jfb15258-bib-0044] Pavlova, A. , Beheregaray, L. B. , Coleman, R. , Gilligan, D. , Harrisson, K. A. , Ingram, B. A. , … Sunnucks, P. (2017). Severe consequences of habitat fragmentation on genetic diversity of an endangered Australian freshwater fish: A call for assisted gene flow. Evolutionary Applications, 10, 531–550. 10.1111/eva.12484. 28616062 PMC5469170

[jfb15258-bib-0045] Peakall, R. , & Smouse, P. E. (2012). GenAlEx 6.5: Genetic analysis in excel. Population genetic software for teaching and research‐‐an update. Bioinformatics, 28, 2537–2539. 10.1093/bioinformatics/bts460. 22820204 PMC3463245

[jfb15258-bib-0046] Peakall, R. , & Smouse, P. E. (2006). Genalex 6: Genetic analysis in excel. Population genetic software for teaching and research. Molecular Ecology Notes, 6, 288–295. 10.1111/j.1471-8286.2005.01155.x. PMC3463245 22820204

[jfb15258-bib-0047] Pritchard, V. L. , Jones, K. , & Cowley, D. E. (2007). Genetic diversity within fragmented cutthroat trout populations. Transactions of the American Fisheries Society, 136, 606–623. 10.1577/T06-038.1.

[jfb15258-bib-0048] Prodöhl, P. A. , Antoniacomi, A. , Bradley, C. , Carlsson, J. , Carvalho, G. R. , Coughlan, J. , … Cross, T. F. (2017). Population genetics and genetic stock identification of anadromous *Salmo trutta* from the Irish Sea and adjacent areas, using microsatellite DNA loci. In G. Harris (Ed.), Sea trout: Science & Management (pp. 69–95). Kibworth Beauchamp, Leicester: Matador.

[jfb15258-bib-0049] Quéméré, E. , Baglinière, J.‐L. , Roussel, J.‐M. , Evanno, G. , McGinnity, P. , & Launey, S. (2016). Seascape and its effect on migratory life‐history strategy influences gene flow among coastal brown trout (*Salmo trutta*) populations in the English Channel. Journal of Biogeography, 43, 498–509. 10.1111/jbi.12632.

[jfb15258-bib-0050] Rochette, N. C. , & Catchen, J. M. (2017). Deriving genotypes from RAD‐seq short‐read data using stacks. Nature Protocols, 12, 2640–2659. 10.1038/nprot.2017.123. 29189774

[jfb15258-bib-0051] Rodríguez‐Ramilo, S. T. , & Wang, J. (2012). The effect of close relatives on unsupervised Bayesian clustering algorithms in population genetic structure analysis. Molecular Ecology Resources, 12, 873–884. 10.1111/j.1755-0998.2012.03156.x. 22639868

[jfb15258-bib-0052] Rousset, F. , 2017. Genepop version 4.7.5.

[jfb15258-bib-0063] Saint‐Pé, K., Leitwein, M., Tissot, L., Poulet, N., Guinand, B., Berrebi, P., Marselli, G., Lascaux, J.‐M., Gagnaire, P.‐A., & Blanchet, S. (2019). Development of a large SNPs resource and a low‐density SNP array for brown trout (*Salmo trutta*) population genetics. BMC Genomics, 20(1). 10.1186/s12864-019-5958-9 PMC6631668 31307373

[jfb15258-bib-0053] Samy, J. K. A. , Mulugeta, T. D. , Nome, T. , Sandve, S. R. , Grammes, F. , Kent, M. P. , … Våge, D. I. (2017). SalmoBase: An integrated molecular data resource for salmonid species. BMC Genomics, 18, 482. 10.1186/s12864-017-3877-1. 28651544 PMC5485693

[jfb15258-bib-0054] Sánchez‐Montes, G. , Ariño, A. H. , Vizmanos, J. L. , Wang, J. , & Martínez‐Solano, Í. (2017). Effects of sample size and full sibs on genetic diversity characterization: A case study of three syntopic Iberian pond‐breeding amphibians. The Journal of Heredity, 108, 535–543. 10.1093/jhered/esx038. 28444211

[jfb15258-bib-0055] Stanier, P. (1985). The granite quarrying industry in Devon and Cornwall part one 1800‐1910. Industrial Archaeology Review, 7, 171–189. 10.1179/iar.1985.7.2.171.

[jfb15258-bib-0056] Toy, H. S. (1934). The Loe bar near Helston. The Geographical Journal, 83, 40. 10.2307/1786663.

[jfb15258-bib-0057] Truett, G. E. , Heeger, P. , Mynatt, R. L. , Truett, A. A. , Walker, J. A. , & Warman, M. L. (2000). Preparation of PCR‐quality mouse genomic DNA with hot sodium hydroxide and tris (HotSHOT). BioTechniques, 29, 52–54. 10.2144/00291bm09. 10907076

[jfb15258-bib-0058] Vincent, B. , & Lawrence, N. (2020). Advisory visit: River Cober. Wild Trout Trust: Cornwall.

[jfb15258-bib-0059] Waples, R. S. (1998). Separating the wheat from the chaff: Patterns of genetic differentiation in high gene flow species. The Journal of Heredity, 89, 438–450. 10.1093/jhered/89.5.438.

[jfb15258-bib-0060] Waples, R. S. , & Hendry, A. P. (2008). Special issue: Evolutionary perspectives on salmonid conservation and management: Evolutionary perspectives on salmonids. Evolutionary Applications, 1, 183–188. 10.1111/j.1752-4571.2008.00035.x. 25567625 PMC3352439

[jfb15258-bib-0066] Westcountry Rivers Troust (2022). Semi & fully quantitative electrofishing surveys ‐ River Camel. Unpublished Report

[jfb15258-bib-0061] Winans, G. A. , Allen, M. B. , Baker, J. , Lesko, E. , Shrier, F. , Strobel, B. , & Myers, J. (2018). Dam trout: Genetic variability in *Oncorhynchus mykiss* above and below barriers in three Columbia River systems prior to restoring migrational access. PLoS One, 13, e0197571. 10.1371/journal.pone.0197571. 29851979 PMC5979028

