# Supplementary material for: A low‐density single nucleotide polymorphism panel for brown trout (Salmo trutta L.) suitable for exploring genetic diversity at a range of spatial scales
Source: J Fish Biol. 2022 Nov 25;102(1):258–70. doi: 10.1111/jfb.15258 (PMC10286751; doi:10.1111/jfb.15258)
Supplement: Supplementary file 1 — FIGURE S1 Example genotype scatter plots for four brown trout SNP assays genotyped on the Fluidigm EP1 platform. Str_68875 shows the three expected genotype clusters with homozygous (red and green) and heterozygous (blue) genotypes, Str_5593 lacks one of the homozygous genotype clusters, Str_59578 is an assay with a high frequency null allele and Str_10193 failed to resolve the three genotype clusters FIGURE S2 Number of single nucleotide polymorphisms per linkage groups for the final panel of 95 Fluidigm assays FIGURE S3 Position and nature (non‐coding, intronic, exonic or untranslated region) on each brown trout linkage group for the final 95 single nucleotide polymorphisms FIGURE S4 Results of COLONY analysis on the ability of the brown trout microsatellite and SNP panels to correctly identify familial relationships. (a) Assignment to full‐sib families and (b) assignment of parentage for 32 simulated trout genotypes. Results were identical for both marker types TABLE S1 Details of DNA pools used for restriction site‐associated DNA (RAD) sequencing with numbers of sequences obtained and the number retained post‐processing through the process_radtags module in STACKS v2.41 (Rochette et al., 2019) TABLE S2 Details of fullsib families simulated using HYBRIDLAB (Nielsen et al., 2001) TABLE S4 Results of COLONY analyses for 30 brown trout from the Great Stour, Kent, UK, genotyped at 18 microsatellite loci and 95 single nucleotide polymorphism assays [file JFB-102-258-s002.docx]

**Supplementary Information for:**

**Development of a low-density single nucleotide polymorphism panel for brown trout (*Salmo trutta* L.)**

**Daniel Osmond, R. Andrew King, Bruce Stockley, Sophie Launey & Jamie R Stevens**

**RADseq**

DNA was extracted from ethanol-stored adipose finclips using Qiagen Blood and Tissue kits for 264 fish from 61 southern British, 25 French rivers and three French hatchery stocks (Table S1) and quantified using Qubit dsDNA HS assays (Life Technologies). We used a modification of traditional restriction site-associated DNA sequencing (pooled-RADseq - Delord et al. 2018) to isolate single nucleotide polymorphisms. Based on previous knowledge of the genetic structuring of English Channel brown trout populations (King et al. 2016, 2020, in preparation; Quéméré et al. 2016), DNA from fish from genetically related rivers were combined, resulting in a total of 20 DNA pools (13 UK pools, seven French pools - six to 18 fish per pool – Table S1). 400ng of DNA was digested at 37°C overnight with *Sbf*I and purified using AmpureXP magnetic beads (Beckman Coulter). Phased P1 adaptors were ligated onto the *Sbf*I cut sites. The use of phasing meant that there were four combinations of adaptor with 0-3 bases between the standard adaptor and the *Sbf*I cut site. Pooled DNA was purified using AmpureXP magnetic beads before fragmentation to an average size of 400 bp using a Covaris E220. Blunt ends were repaired and adenylated using the Nextflex End repair and adenylation kit (Perkin Elmer) prior to ligation of the P2 adaptor. The library was PCR amplified for 14 cycles prior to quantification and validation using a D1000 HS screentape on a Tapestation (Agilent). RAD libraries were sequenced on an Illumina HiSeq 2500 in rapid run mode (250 bp paired-end sequencing).

**Table S1** Details of DNA pools used for restriction site-associated DNA (RAD) sequencing with numbers of sequences obtained and the number retained post-processing through the process_radtags module in STACKS v2.41 (Rochette et al. 2019)

| Pool | Region | N_DNA_ | Rivers | Nseq | Nseq post |
| --- | --- | --- | --- | --- | --- |
| GB01A | south Wales | 8 | Taf, Nevern, Tawe, Tywi | 2540604 | 1188704 |
| GB01B | Bristol Channel | 16 | Severn, Wye, Bristol Avon, Doniford, East Lyn, Taw, Torridge, Tone | 9635782 | 5631559 |
| GB02 | Cornwall | 17 | Allen, Caerhays, Camel, Fal, Gannel, Gweek, Kennal, Tresillian | 7391142 | 5808517 |
| GB03 | Metal | 18 | Crowlas, Hayle, Red River, Trevaylor | 8094550 | 5494434 |
| GB04A | South Cornwall | 14 | Fowey, Lerryn, East Looe, West Looe | 5137448 | 4085905 |
| GB04B | Tamar Estuary/South Hams | 17 | Erme, Lynher, Plym, Seaton, Tamar, Tavy, Yealm | 12679578 | 8759074 |
| GB04C | South Devon | 14 | Dart, Devon Avon, Teign | 11533848 | 6633887 |
| GB05 | EOA | 16 | Exe, Otter, Axe | 11777720 | 8042679 |
| GB06 | Brit | 6 | Brit | 5513196 | 4466178 |
| GB07 | Hants Basin | 17 | Frome, Itchen, Meon, Piddle, Test, Wallington | 10651912 | 6669425 |
| GB08 | Hants Avon | 10 | Hants Avon | 8083398 | 5504386 |
| GB09 | SE | 18 | Adur, Arun, Dour, Eastern Rother, Great Stour, Sussex Ouse | 7158852 | 5369238 |
| GB10 | Thames/Norfolk | 14 | Burn, Glaven, Medway, Stiffkey, Thames | 10885514 | 7829916 |
| FR01 | Brittany 1 | 15 | Aber Wrac’h, Flèche, Horn, Guillec, Kérallé | 7167266 | 5686594 |
| FR02 | Brittany 2 | 12 | Yar, Léguer, Trieux | 5764332 | 4679384 |
| FR03 | West Cotentin | 12 | Couesnon, Sée, Sienne, Sélune | 10840210 | 7694598 |
| FR04 | Lower Normandy 1 | 12 | Vire, Saire, Orne, Seulles | 5590172 | 4448843 |
| FR05 | Lower Normandy 2 | 10 | La Touques, La Dives | 5990484 | 4179747 |
| FR06 | Upper Normandy | 18 | Sanne, Scie, Varennes, Béthune, Eaulne, Yères, Bresle | 8265552 | 7248541 |
| FR07 | French hatchery | 12 | Three hatchery stocks | 11252596 | 7336120 |

**SNP Discovery & Filtering**

Adaptor sequences and phasing were removed using cutadapt v2.5 (Martin 2011) and custom scripts (Paul O’Neill, personal communication), respectively. RADseq data was processed and analysed using STACKS v2.41 (Rochette et al. 2019). Raw reads were demultiplexed and trimmed to 150 bp in length using process_radtags. Preliminary analysis showed that three population pools (GB01A, GB01B and GB04C) had either a low sequence coverage or high levels of missing data (>25%). Therefore, these libraries were removed from all subsequent analyses. Initial optimization of *M* (number of mismatches allowed between stacks within individuals) and *n* (number of mismatches allowed between stacks between individuals) parameters was conducted following the 80% method of Paris et al. (2017) using all retained libraries. RAD loci were built using optimised parameters (*M*=1, *n*=2, *m*=3 (default)) using the denovo_map.pl pipeline. The populations module was run to filter RAD loci for subsequent Fluidigm assay design. To be scored, SNPs had to be found in at least 90% of the libraries (-r 0.9) and have a minimum allele frequency of at least 0.15 (--min-maf 0.15). A whitelist of RAD loci containing only a single SNP were extracted from the ‘populations.sumstats.tsv’ output. The populations module was run a second time using this whitelist to output data for these loci only. In addition, we generated vcf and fasta files from this analysis. Information on the sequence and length of each RAD locus was extracted from the fasta file and the position and nature of each polymorphism were extracted from the vcf file.

The STACKS-filtering process resulted in a total of 7653 RAD loci containing a single variable nucleotide. To aid primer design for subsequent analysis on the Fluidigm EP1 system, loci were filtered further by removing all loci where the SNP was present in the first or last 60 bases of the locus sequence, resulting in the rejection of 2123 loci, leaving a dataset of 5530 loci.

**Non-RADseq derived polymorphisms**

To the RADseq-derived loci we added sequence from three additional genomic regions. The first was an indel polymorphism. Primers for a microsatellite marker, One102, amplify two loci with non-overlapping size ranges, designated One102a and One102b, in trout. One102a has been shown to possess only two alleles differing in size by three base pair (Paris et al. 2015; King et al. 2020; Prodöhl et al. 2020). Sequences for two addition SNP markers were also included - a nonsynonymous substitution in exon 2 of the *vestigial-like family member 3* (*vgll3*) gene and a C/G polymorphism in an intron of the *metallothionein B* (*metB*) gene. One102a was amplified using the primers of Keenan et al. (2013), Exon 2 of the *vgll3* gene was amplified using primers vgll3F2 and vgll3rev (King & Stevens 2021) and metB was amplified using primers metB-v2-F (GGCTCAAGATGGTACAACCAAT) and metB-v2-R (CGTCTGTCCTGACGCTATAAAA).

Amplifications were performed in a 10 μL volume, containing 5 μL of HotStar Taq Master Mix Kit (Qiagen), 0.15 μM each primer and 1 μL of extracted DNA. PCR cycling conditions were 95˚C for 5 min, followed by 35 cycles of 94ºC for 30 s, 56ºC (One102a), 58ºC (*vgll3* & *metB*) for 30 s, 72ºC for 40 s and a final extension at 72ºC for 10 min.

Products from One102a were purified using QIAquick PCR Purification Kits (Qiagen) and cloned using the TOPO TA Cloning® kit (Invitrogen) following the manufacturer’s instructions. Cloned inserts were amplified using M13 primers. PCR reactions were carried out in total volume of 15 μl consisting of 7.5 μL of HotStar Taq Master Mix Kit, 1.5 μg BSA, 0.1 μM each forward and reverse primer and 1 μl of overnight-grown colony suspension. After an initial denaturing step at 94°C for 2 min 30 s, amplification proceeded for 35 cycles at 94°C for 30 s, 55°C for 30 s, 72°C for 45 s and a final extension at 72°C for 10 min.

For Sanger sequencing, Exonuclease I and Antarctic Phosphotase (both New England Biolabs) were used to purify PCR products (37ºC for 45 min and 80ºC for 15 min). Products were sequenced in forward and reverse directions on a 3730xl Genetic Analyzer (Applied Biosystems) by EUROFINS (Ebersberg, Germany).

**BLAST analysis**

We aligned the sequences from 1070 randomly chosen RAD loci against the reference brown trout genome using the NCBI blastn portal (https://blast.ncbi.nlm.nih.gov/Blast.cgi). We retained loci that aligned strongly to only a single linkage group, noting linkage group and position on that group. We then used the Genome Browser facility of SalmoBase (https://salmobase.org, Samy et al. 2017) to determine if each RAD locus represented coding or non-coding sequence. For coding sequence, we recorded whether the polymorphic base was found in an intron or exon.

**Assay design**

Loci were then ranked for use, with preference for high accuracy of matches, no full-length secondary hits and high heterozygosity in the 17 retained pooled libraries. The sequence for 162 RAD loci were submitted to the Fluidigm D3 Assay Design platform, with 157 being returned as suitable for primer design. A random set of 156 assays were synthesised by Fluidigm. These loci were initially tested against a panel of brown trout samples multiple English Channel/La Manche rivers.

**Supplementary Figure 1** Example genotype scatter plots for four brown trout SNP assays genotyped on the Fluidigm EP1 platform. Str_68875 shows the three expected genotype clusters with homozygous (red & green) and heterozygous (blue) genotypes, Str_5593 lacks one of the homozygous genotype clusters, Str_59578 is an assay with a high frequency null allele and Str_10193 failed to resolve the three genotype clusters

**
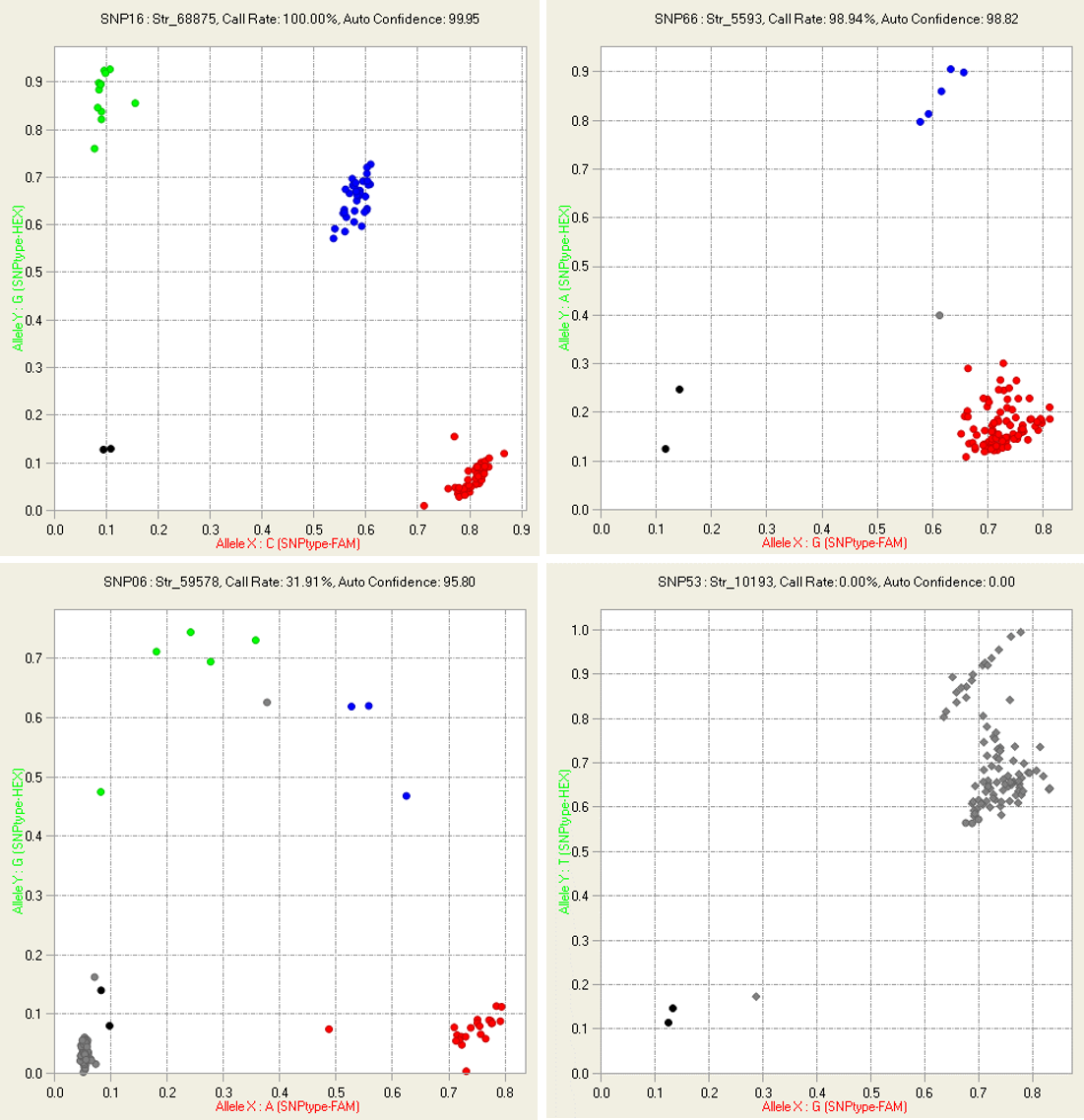
**

**Supplementary Figure 2** Number of single nucleotide polymorphisms per linkage groups for the final panel of 95 Fluidigm assays.


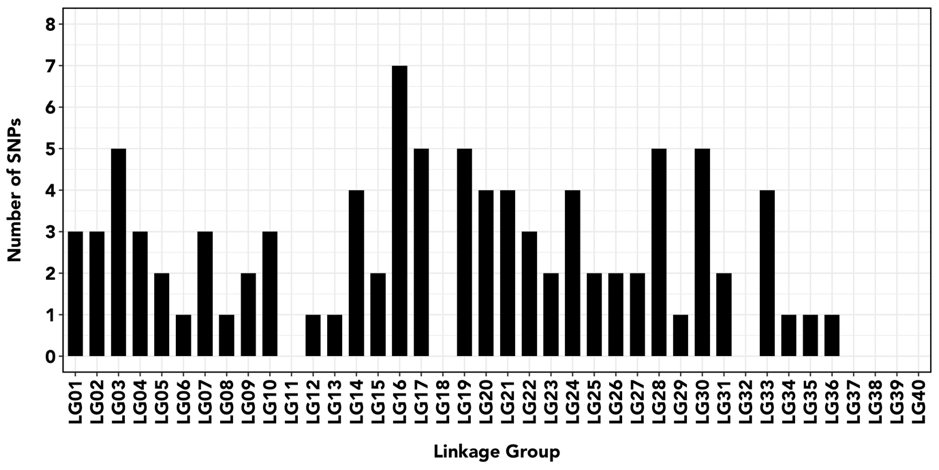


**Supplementary Figure 3** Position and nature (non-coding, intronic, exonic or untranslated region) on each brown trout linkage group for the final 95 single nucleotide polymorphisms.


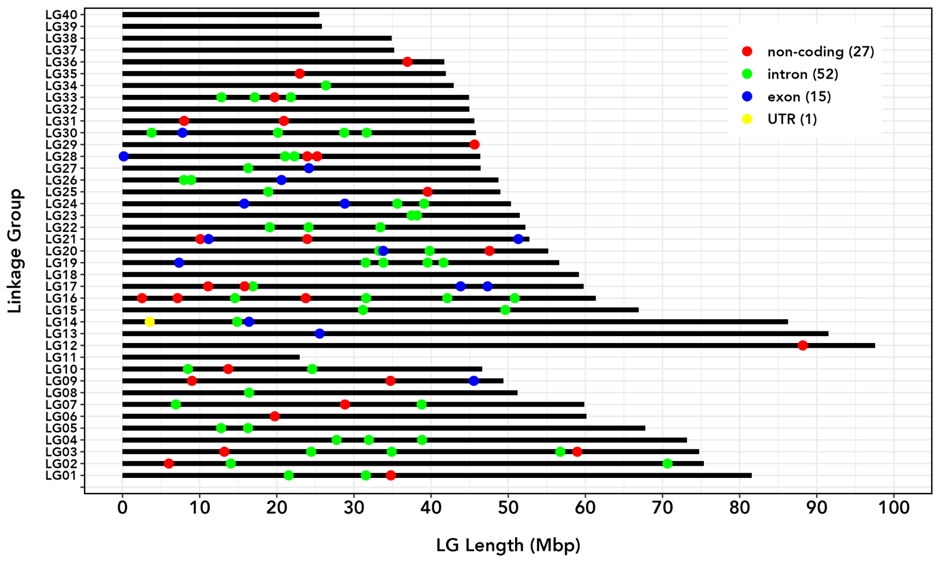


**Supplementary Figure 4** Results of COLONY analysis on the ability of the brown trout microsatellite and SNP panels to correctly identify familial relationships. a) assignment to fullsib families and b) assignment of parentage for 32 simulated trout genotypes. Results were identical for both marker types.


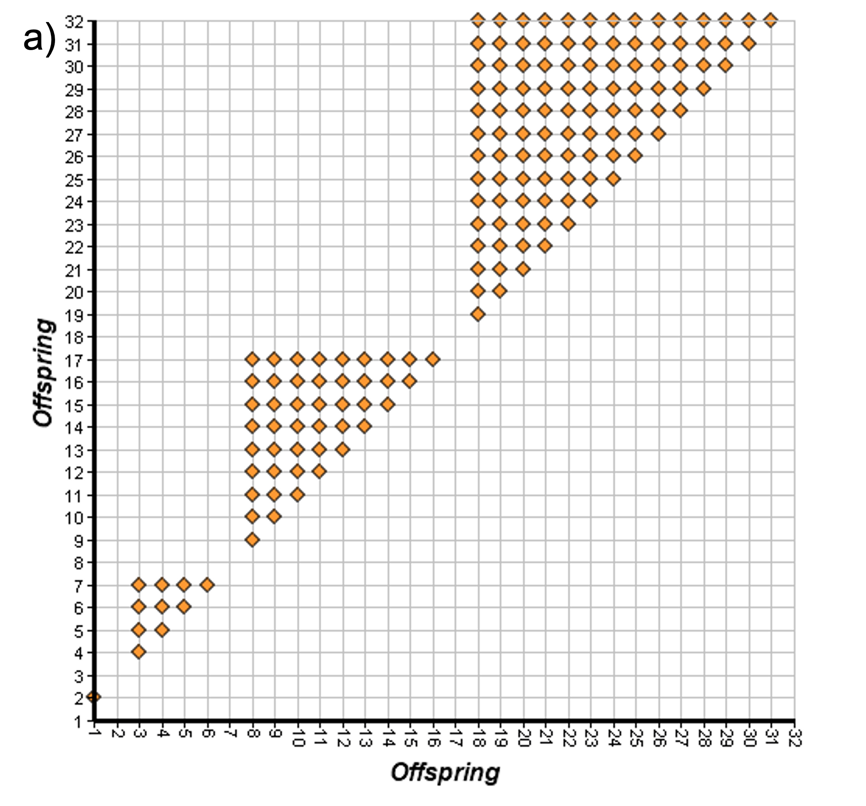

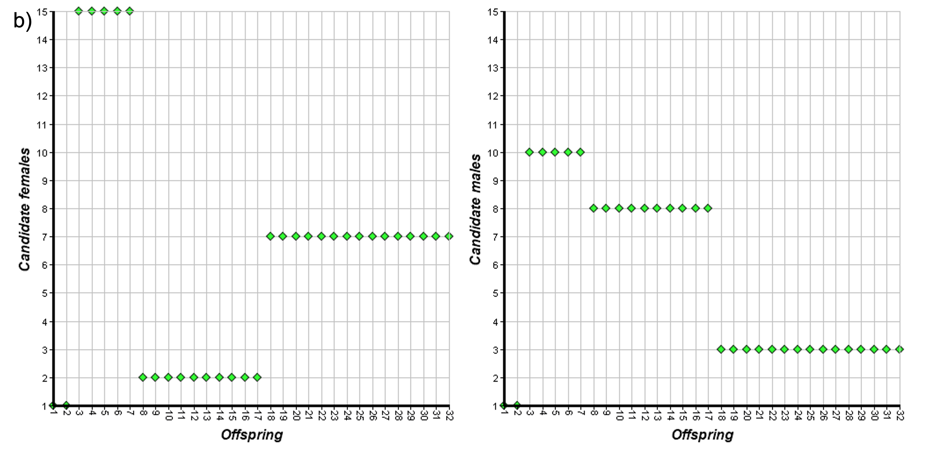


**Supplementary Table 2** Details of fullsib families simulated using HYBRIDLAB (Nielsen et al. 2001).

|  | **Male genotype** | **Female genotype** | **N_fullsibs_** |
| --- | --- | --- | --- |
| Family 1 | M1 | F1 | 2 |
| Family 2 | M10 | F15 | 5 |
| Family 3 | M8 | F2 | 10 |
| Family 4 | M3 | F7 | 15 |

**Supplementary Table 4** Results of COLONY analyses for 30 brown trout from the Great Stour, Kent, UK genotyped at 18 microsatellite loci and 95 single nucleotide polymorphism assays.

| **Data set** | **Family** | **Probability of Inclusion^a^** | **Probability of Exclusion^b^** | **Member 1** | **Member 2** | **Member 3** | **Member 4** | **Member 5** | **Member 6** | **Member 7** |
| --- | --- | --- | --- | --- | --- | --- | --- | --- | --- | --- |
| Microsatellite | 1 | 0.9933 | 0.9933 | GST01 | GST23 |  |  |  |  |  |
|  | 2 | 1 | 1 | GST02 |  |  |  |  |  |  |
|  | 3 | 1 | 1 | GST03 | GST05 | GST09 | GST12 | GST15 | GST17 | GST29 |
|  | 4 | 1 | 1 | GST04 |  |  |  |  |  |  |
|  | 5 | 1 | 1 | GST06 |  |  |  |  |  |  |
|  | 6 | 0.2946 | 0.2946 | GST07 | GST13 | GST19 | GST22 |  |  |  |
|  | 7 | 1 | 0.9965 | GST08 |  |  |  |  |  |  |
|  | 8 | 1 | 1 | GST10 |  |  |  |  |  |  |
|  | 9 | 1 | 0.411 | GST11 | GST30 |  |  |  |  |  |
|  | 10 | 1 | 1 | GST14 |  |  |  |  |  |  |
|  | 11 | 1 | 1 | GST16 | GST21 |  |  |  |  |  |
|  | 12 | 1 | 1 | GST18 | GST20 |  |  |  |  |  |
|  | 13 | 1 | 0.9965 | GST24 |  |  |  |  |  |  |
|  | 14 | 1 | 0.9796 | GST25 |  |  |  |  |  |  |
|  | 15 | 0.7665 | 0.4103 | GST26 | GST27 |  |  |  |  |  |
|  | 16 | 1 | 1 | GST28 |  |  |  |  |  |  |
|  |  |  |  |  |  |  |  |  |  |  |
| SNP | 1 | 1 | 1 | GST01 | GST23 |  |  |  |  |  |
|  | 2 | 1 | 1 | GST02 |  |  |  |  |  |  |
|  | 3 | 0.9214 | 0.9214 | GST03 | GST05 | GST09 | GST12 | GST15 | GST17 | GST29 |
|  | 4 | 1 | 1 | GST04 |  |  |  |  |  |  |
|  | 5 | 1 | 1 | GST06 |  |  |  |  |  |  |
|  | 6 | 1 | 1 | GST07 | GST13 | GST19 | GST22 |  |  |  |
|  | 7 | 1 | 1 | GST08 |  |  |  |  |  |  |
|  | 8 | 1 | 0.9981 | GST10 |  |  |  |  |  |  |
|  | 9 | 1 | 0.9978 | GST11 | GST30 |  |  |  |  |  |
|  | 10 | 1 | 1 | GST14 |  |  |  |  |  |  |
|  | 11 | 1 | 0.9981 | GST16 | GST21 |  |  |  |  |  |
|  | 12 | 1 | 1 | GST18 | GST20 |  |  |  |  |  |
|  | 13 | 1 | 1 | GST24 |  |  |  |  |  |  |
|  | 14 | 1 | 1 | GST25 |  |  |  |  |  |  |
|  | 15 | 1 | 0.5254 | GST26 |  |  |  |  |  |  |
|  | 16 | 1 | 0.5254 | GST27 |  |  |  |  |  |  |
|  | 17 | 1 | 1 | GST28 |  |  |  |  |  |  |

^a^ – probability that all individuals of a given family are full sibs

^b^ – probability that all individuals of a given family are full sibs and that no other individuals are full sibs within that family

**References**

Delord C, Lassalle G, Oger A, Barloy, D Coutellec M-A, Delcamp A et al. (2018) A cost-and-time effective procedure to develop SNP markers for multiple species: A support for community genetics. Methods in Ecology & Evolution 9, 1959-1974

Keenan K, Bradley CR, Magee JJ et al. (2013) Beaufort trout MicroPlex: a high-throughput multiplex platform comprising 38 informative microsatellite loci for use in resident and anadromous (sea trout) brown trout *Salmo trutta* genetic studies. Journal of Fish Biology, **82**, 1789–1804

King RA, Hillman R, Elsmere P, Stockley B & Stevens JR (2016) Investigating patterns of straying and mixed stock exploitation of sea trout, *Salmo trutta,* in rivers sharing an estuary in south-west England. Fisheries Management & Ecology 23, 376–389

King RA, Stockley B & Stevens JR (2020) Small coastal streams—Critical reservoirs of genetic diversity for trout (*Salmo trutta* L.) in the face of increasing anthropogenic stressors. Ecology & Evolution 10, 5651–5669

King RA & Stevens JR (2021) Development of SNP markers derived from RAD sequencing for Atlantic salmon (*Salmo salar* L.) inhabiting the rivers of southern England. Conservation Genetics Resources **13**, 369-373

Martin M (2011) Cutadapt removes adapter sequences from high-throughput sequencing reads. *EMBnet.journal* **17**, 10

Nielsen, E.E., Hansen, M.M., Bach, L.A., 2001. Looking for a needle in a haystack: discovery of indigenous salmon in heavily stocked populations. Conservation Genetics **2**, 219–232

Paris JR, King RA & Stevens JR (2015) Human mining activity across the ages determines the genetic structure of modern brown trout (*Salmo trutta* L.) populations. Evolutionary Applications **8**, 573-585

Paris JR, Stevens JR, Catchen JM (2017) Lost in parameter space: a road map for stacks. Methods in Ecology Evolution **8**, 1360–1373

Prodöhl PA, Ferguson A, Bradley CR et al. (2020) Impacts of acidification on brown trout *Salmo trutta* populations and the contribution of stocking to population recovery and genetic diversity. Journal of Fish Biology **95**, 719-742

Quéméré E, Baglinière J-L, Roussel J-M, Evanno G, McGinnity P & Launey S (2016) Seascape and its effect on migratory life-history strategy influences gene flow among coastal brown trout (*Salmo trutta*) populations in the English Channel. Journal of Biogeography 43, 498-509

Rochette NC, Rivera-Colón AG & Catchen JM (2013) Stacks 2: Analytical methods for paired-end sequencing improve RADseq-based population genomics. Molecular Ecology 28, 4737–4754

Samy JKA, Mulugeta TD, Nome T, Sandve SR, Grammes F, Kent MP et al. (2017) SalmoBase: an integrated molecular data resource for Salmonid species. BMC Genomics 18, 482. https://doi. org/10.1186/s12864-017-3877-1
